# 

**Published:** 1875-10

**Authors:** 


### CONTENTS:

### ORIGINAL COMMUNICATIONS.

ARTICLE I.—Probabilities. By Dr. G. V. Black.......... 241

ARTICLE II.—Mechanical Dentistry. By Dr. O. Willson.......... 250

### SELECTED ARTICLES.

ARTICLE III.—American Dental Association.......... 260

ARTICLE IV.—A Few Facts from Salter. By J. Edw. Line, D. D. S.......... 268

ARTICLE V.—Treatment of Exposed Pulps in Filling Teeth. (Concluded.) By Samuel Welchens, D. D. S.......... 276

### EDITORIAL, ETC.

The Progress of Dentistry.......... 281
The American Academy of Dental Surgery.......... 285

### BIBLIOGRAPHICAL.

The Histology and Histochemistry of Man.......... 285
The Cholera Epidemic of 1873 in the United States.......... 286
Transactions of the College of Physicians, of Philadelphia.......... 286
Transactions of the Medical and Chirurgical Faculty of Maryland.......... 286

### MONTHLY SUMMARY.

Dispensing with Rain.......... 287

Ether vs. Chloroform.......... 288

Effects of the Inhalation of Chloroform during Parturition, on the Fœtus.......... 288

